# The influence of preoperative dialysis on survival after continuous-flow left ventricular assist device implantation

**DOI:** 10.1093/icvts/ivab357

**Published:** 2021-12-30

**Authors:** Harveen K Lamba, Fadi I Musfee, Subhasis Chatterjee, Ajith P Nair, Andrew B Civitello, Leo Simpson, O H Frazier, George V Letsou

**Affiliations:** 1 Division of Cardiothoracic Transplantation and Circulatory Support, Baylor College of Medicine, Houston, TX, USA; 2 Department of Cardiopulmonary Transplantation and the Center for Cardiac Support, Texas Heart Institute, Houston, TX, USA; 3 Department of Epidemiology, Human Genetics, and Environmental Sciences, UT Health School of Public Health, Houston, TX, USA; 4 Michael E. DeBakey Department of Surgery, Baylor College of Medicine, Houston, TX, USA

**Keywords:** Heart failure, Left ventricular assist device, End-stage renal disease, Haemodialysis

## Abstract

**OBJECTIVES:**

Dialysis is considered a contraindication to continuous-flow left ventricular assist device (CF-LVAD) implantation. We evaluated clinical outcomes and survival in carefully selected, low-risk patients with renal failure who required dialysis before CF-LVAD implantation.

**METHODS:**

We extracted medical record data of patients who underwent CF-LVAD placement at our centre between 1 January 2006 and 31 August 2017, with 2 clinical scenarios: those who required long-term (>14 days) dialysis and those who required short-term (≤14 days) dialysis immediately before implantation. Demographic, clinical and intraoperative characteristics and survival outcomes were assessed.

**RESULTS:**

Of 621 patients who underwent CF-LVAD implantation during the study period, 31 underwent dialysis beforehand. Of these, 17 required long-term dialysis (13 haemodialysis, 4 peritoneal dialysis), and 14 underwent short-term haemodialysis. Compared with the long-term dialysis patients, the short-term dialysis patients were more likely to be Interagency Registry for Mechanically Assisted Circulatory Support profile 1–2 (92.9% vs 70.6%; *P *<* *0.001), to have needed preoperative mechanical circulatory support (78.6% vs 70.6%; *P *<* *0.01) and to have higher in-hospital mortality (85.7% vs 29.4%; *P *=* *0.01). Patients stable on long-term dialysis had acceptable overall survival and markedly better 6-month and 1-year survival than those with short-term dialysis before implantation (64.7% vs 14.3% and 58.8% vs 7.1%, respectively; *P *<* *0.001).

**CONCLUSIONS:**

Carefully selected patients who are stable on long-term dialysis have acceptable survival rates after CF-LVAD implantation. Patients with acute renal failure had much poorer outcomes than those with chronic end-stage renal disease.

**Figure:**
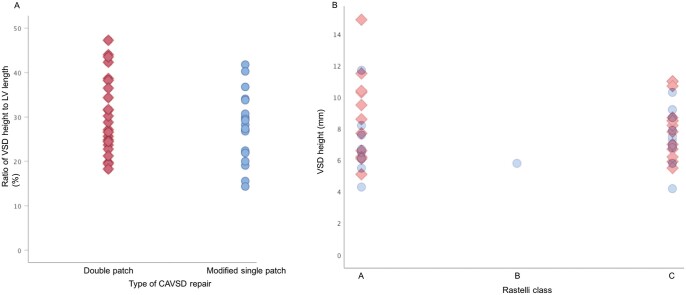

## INTRODUCTION

Continuous-flow left ventricular assist devices (CF-LVADs) are commonly used in patients with end-stage heart failure as a bridge to cardiac transplant or as destination therapy for those who do not qualify for cardiac transplant. Although the criteria for CF-LVAD eligibility are not as strict as those for cardiac transplant, patient selection and timing for CF-LVAD implantation remain controversial, especially given the cost and potential complications.

End-organ failure, particularly renal dysfunction, is a major complication of heart failure that increases morbidity and mortality [1, 2]. Approximately 40% of patients with end-stage renal disease (ESRD) have heart failure, and 37% of patients with ESRD die from heart failure [3]. Because CF-LVAD implantation in these patients is associated with high perioperative mortality [4], dialysis has been considered a contraindication for CF-LVAD implantation. However, several studies have shown improved renal function after CF-LVAD implantation [5–7], raising the question of whether CF-LVAD implantation could be justified in patients with ESRD.

We investigated our single-centre experience with all patients who required dialysis before heart transplant. We examined 2 distinct groups: patients who required dialysis for >14 days before CF-LVAD implantation and those who required dialysis for ≤14 days before implantation. We compared clinical outcomes and postoperative survival between these 2 patient groups.

## METHODS

### Ethics statement

The study was approved by the Baylor College of Medicine Institutional Review Board (approval # H-38751, dated 24 November 2016), and informed consent was obtained from each patient before CF-LVAD implantation.

### Patients

We retrospectively reviewed the medical records of patients who underwent initial implantation of either a HeartMate II (Thoratec Corporation, Pleasanton, CA, USA) or HeartWare (HeartWare Inc., Framingham, MA, USA) CF-LVAD at our institution between 1 January 2006 and 31 August 2017, either as a bridge to transplant or as destination therapy, depending on the patient’s eligibility for cardiac transplant. All patients undergoing CF-LVAD implantation at our institution are evaluated by a multidisciplinary committee, and the patient’s need for long-term or short-term dialysis is taken into consideration before surgery is approved. Patients who required dialysis before CV-LVAD implantation were included in the study. Patients undergoing exchange of an existing CF-LVAD were excluded from the study.

Patients requiring dialysis presented with 2 phenotypes. One group of patients had a history of chronic renal insufficiency requiring dialysis. The other group required initiation of dialysis in the days or weeks before CF-LVAD implantation because of acute deterioration in renal function. Thus, we divided patients into 2 groups: those who required long-term dialysis (for >14 days) and those who required short-term dialysis (for ≤14 days) before the first CF-LVAD implantation.

### Data analysis

Recorded variables included demographic (patient age, sex, weight, body mass index) and clinical (dialysis type, aetiology of heart failure, comorbid conditions, hematological and biochemical values, mechanical circulatory support before CF-LVAD implantation, haemodynamic data and intraoperative data) variables. Comorbid diagnoses and perioperative outcome variables adhered to the definitions of the Interagency Registry for Mechanically Assisted Circulatory Support (INTERMACS) [8], unless otherwise stated. We assessed survival in both patient groups.

For continuous variables, data are expressed as the median [interquartile range (IQR) p25–p75]. Categorical variables are presented as proportions. Differences between groups were assessed by using Fisher’s exact test for categorical variables, independent Student’s *t*-test for normally distributed continuous variables, and Mann–Whitney’s *U*-test for non-normally distributed continuous variables.

Survival analysis was performed by using the Kaplan–Meier method. Survival results by haemodialysis status and CF-LVAD insertion indication were compared between the 2 groups using the log-rank test. The follow-up period continued through the end of the retrospective review (31 October 2017). A Kaplan–Meier curve of INTERMACS survival data is provided as a reference comparator. Log-rank testing was not used to compare the INTERMACS survival data with our patient cohorts’ survival data, due to the size discrepancies between the groups.

Statistical analyses were performed by using IBM SPSS Statistics for Macintosh, version 25 (SPSS, Inc, Armonk, NY, USA). A *P*-value of <0.05 was considered statistically significant.

## RESULTS

### Baseline clinical characteristics

Among the 621 patients who underwent CF-LVAD implantation at our institution during the study period, 31 were on dialysis before CF-LVAD implantation: 17 were on long-term dialysis (>14 days) before implantation and 14 were on short-term dialysis (≤14 days) before implantation. The CF-LVADs were implanted as a bridge to transplant in 10 (58.8%) of the 17 patients on long-term dialysis and in all 14 (100.0%) of the patients on short-term dialysis.

For the 17 patients on long-term dialysis, the time on dialysis before CF-LVAD placement ranged from 15 to 3285 days. Fifteen patients had intermittent haemodialysis or intermittent peritoneal dialysis for at least 1 month before CF-LVAD placement; the remaining 2 patients were on continuous veno-venous hemofiltration for 15 and 16 days, respectively, before implantation. These 2 patients had a history of chronic renal insufficiency and, although they had never been on dialysis, they did require it before CF-LVAD implantation, due to renal disease progression. Given their history of underlying chronic renal insufficiency, a consulting nephrologist determined that neither patient had recoverable kidney function. These 2 patients were more similar phenotypically to patients in the long-term dialysis group than in the short-term dialysis group, who had no other history of renal insufficiency and required continuous renal replacement therapy due to advanced heart failure. For these reasons, we included the 2 patients in the long-term dialysis group. Time from ESRD diagnosis was not assessed in patients on short-term dialysis, as they underwent dialysis for acute renal failure related to cardiogenic shock and did not have a previous ESRD diagnosis.

Baseline demographic and preoperative patient characteristics are summarized in Table 1. The long-term dialysis group comprised 16 men and 1 woman; 13 had undergone haemodialysis and 4 had undergone peritoneal dialysis. The median age was 52.0 years (IQR 46.5–63.0 years). Heart failure aetiology was ischaemic in 8 patients (47.1%). The cause of renal failure was missing for 5 of the patients, which is a limitation of retrospective chart review. For the remaining patients, aetiology varied and included cardiorenal syndrome, diabetes, contrast- or chemotherapy-induced acute tubular necrosis, cardiorenal syndrome, immunoglobulin A nephropathy and hypertensive nephrosclerosis.

**Table 1:** Demographic and preoperative characteristics of patients on dialysis who underwent continuous-flow left ventricular assist device implantation

Variable	Long-term dialysis^a^ (*N *=* *17)	Short-term dialysis^a^ (*N *=* *14)	*P-*Value
Age (years)	52.0 (46.5–63.0)	54.5 (42.0–59.0)	0.33
BMI (kg/m^2^)	25.7 (22.6–31.0)	28.3 (23.2–31.2)	0.93
Male sex	16 (94.1)	12 (85.7)	0.53
INTERMACS 1	5 (29.4)	9 (64.3)	<0.001
INTERMACS 2	8 (47.1)	5 (35.7)	<0.001
INTERMACS 3	2 (11.8)	0	<0.001
INTERMACS 4 or 5	2 (11.8)	0	<0.001
Ischaemic aetiology	8 (47.1)	4 (28.6)	0.72
Bridge to transplant status	10 (58.8)	7 (50.0)	0.41
Previous cardiac surgery	7 (41.1)	6 (42.9)	0.73
Diabetes mellitus	6 (35.3)	3 (21.4)	0.24
Hypertension	1 (5.9)	5 (35.7)	0.11
Myocardial infarction	1 (5.9)	1 (7.1)	0.63
Previous mechanical circulatory support	12 (70.6)	11 (78.6)	0.01

Continuous variables are expressed as median (IQR p25–p75); categorical variables are expressed as *n* (%).

a Long-term dialysis is that lasting more than 14 days; short-term dialysis is that lasting 14 days or less.

BMI: body mass index; INTERMACS: Interagency Registry for Mechanically Assisted Circulatory Support; IQR: interquartile range.

The short-term dialysis group comprised 12 men and 2 women, all on continuous veno-venous haemofiltration. The median age was 54.5 years (IQR 42.0–59.0 years). Aetiology of heart failure was ischaemic in 4 patients (28.6%). None of these characteristics differed significantly between the 2 groups.

Of the 17 patients who underwent long-term dialysis, 12 (70.6%) were classified as INTERMACS 1 or 2, compared with 13 (92.9%) of the 14 patients who underwent short-term dialysis (*P *<* *0.001). Preoperative mechanical circulatory support was required by 12/17 patients (70.6%) in the long-term dialysis group and by 11/14 patients (78.6%) in the short-term dialysis group (*P *<* *0.01). Preoperative mechanical circulatory support methods included intra-aortic balloon pump, TandemHeart percutaneous ventricular assist device (Cardiac Assist Inc., Pittsburgh, PA, USA), CentriMag (Thoratec, Pleasanton, CA, USA), Impella pump (Abiomed, Danvers, MA, USA) and venous-arterial extracorporeal membrane oxygenation.

Complete haematological, biochemical and haemodynamic data for these patients before CF-LVAD implantation are provided in Table 2. Creatinine level and platelet count were significantly higher, and WBC count, bilirubin and mean right atrial pressure were significantly lower in the long-term dialysis group, compared with the short-term dialysis group. There were no statistically significant differences in intraoperative characteristics between the 2 patient groups (Table 3).

**Table 2:** Laboratory and haemodynamic values of patients on dialysis before continuous-flow left ventricular assist device implantation

Parameter	Long-term dialysis^a^ (*N *=* *17)	Short-term dialysis^a^ (*N *=* *14)	*P-*Value
BUN (mg/dl)	30.0 (22.0–39.0)	28.5 (18.3–45.3)	0.79
Creatinine (mg/dl)	2.1 (1.5–3.9)	1.5 (1.2–2.2)	<0.001
Sodium (mEQ/l)	136.0 (135.0–138.8)	135.5 (130.5–138.0)	0.78
Haemoglobin (g/dl)	10.4 (9.7–12.0)	9.8 (8.6–10.7)	0.006
WBC count, × 10^3^ (mm^3^)	10.3 (5.9–12.5)	14.7 (11.0–19.5)	0.001
Platelet count, × 10^9^/l	163.0 (101.0–248.0)	132.2 (69.3–151.3)	0.002
Serum albumin (g/l)	3.6 (3.0–3.9)	3.4 (2.9–10.7)	0.47
Total bilirubin (mg/dl)	0.7 (0.5–1.4)	3.3 (1.9–14.1)	0.001
AST (U/l)	33.5 (27.8–46.5)	46.5 (27.3–84.0)	0.19
ALT (U/l)	31.5 (21.8–44.5)	29.0 (22.3–78.5)	0.85
Left ventricular end diastolic dimension (cm)	6.2 (5.7–7.1)	6.2 (4.9–7.9)	0.38
Cardiac index (l/min/m^2^)	1.9 (1.5–2.4)	1.7 (1.3–2.2)	0.99
Mean right atrial pressure (mmHg)	13.0 (12.0–21.0)	15.0 (12.5–22.0)	0.02
PCWP (mmHg)	32.0 (20.5–37.3)	26.0 (23.0–35.0)	0.37
Mean pulmonary artery pressure (mmHg)	46.0 (32.8–50.8)	38.0 (33.0–50.8)	0.06
Peripheral vascular resistance (Wood units)	3.3 (2.2–4.4)	3.5 (1.5–4.2)	0.95

Values are expressed as median (IQR p25–p75).

a Long-term dialysis is that lasting more than 14 days; short-term dialysis is that lasting 14 days or less.

ALT: alanine aminotransferase; AST: aspartate aminotransferase; BUN: blood urea nitrogen; IQR: interquartile range; IQR: interquartile range; PCWP: pulmonary capillary wedge pressure; WBC: white blood cell.

**Table 3:** Intraoperative characteristics of patients on dialysis before continuous-flow left ventricular assist device implantation

Variable	Long-term dialysis^a^ (*N *=* *17)	Short-term dialysis^a^ (*N *=* *14)	*P*-Value
CF-LVAD device			
HeartMate II	16 (94.1)	13 (92.9)	0.81
HeartWare	1 (5.9)	1 (7.1)	0.81
Cardiopulmonary bypass time (min)	105.5 (77.8–148.8)	137.5 (76.0–215.8)	0.21
Concomitant procedure	7 (41.2)	5 (35.7)	0.23

Continuous variables are expressed as median (IQR p25–p75); categorical variables are expressed as *n* (%).

a Long-term dialysis is that lasting more than 14 days; short-term dialysis is that lasting 14 days or less.

CF-LVAD: continuous-flow left ventricular assist device; IQR: interquartile range.

### Survival and outcome analysis

#### Long-term dialysis group

In the long-term dialysis group, 4 of 17 survived to heart transplant after receiving CF-LVAD support for a median 113.5 days (IQR 20.0–209.5 days). A fifth patient was listed for combined heart and kidney transplant but was delisted after being diagnosed with multiple myeloma; to date, this patient has remained on CF-LVAD support for >655 days. Median survival after CF-LVAD implantation was 0.6 years (IQR 0.2–1.8 years) in the 13 patients who remained on CF-LVAD support in the long-term dialysis group. Overall, the patients who underwent CF-LVAD implantation as bridge to transplant (*n *=* *10) had a median survival time of 1.5 years (IQR 0.5–1.8 years), whereas those who underwent CF-LVAD implantation as destination therapy (*n *=* *7) had a median survival of 0.5 years (IQR 0.2–2.7 years).

**Figure 1:**
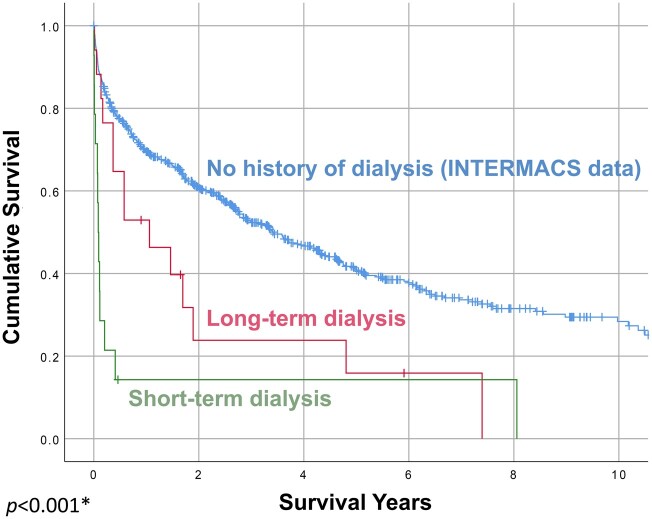
Survival after continuous-flow left ventricular assist device implantation, by preoperative haemodialysis status. The short-term (≤14 days) and long-term (>14 days) dialysis results differed statistically by log-rank analysis. The blue line represents continuous-flow left ventricular assist device survival in all patients from the Interagency Registry for Mechanically Assisted Circulatory Support registry during the same timeframe as our study; it is included for reference and was not compared statistically to the long-term and short-term dialysis survival curves. *P <* *0.001, log-rank test comparing long-term dialysis versus short-term dialysis. INTERMACS: Interagency Registry for Mechanically Assisted Circulatory Support.

Twelve patients in the long-term dialysis group survived to discharge, and 5 did not, resulting in an in-hospital mortality rate of 29.4%. The overall 6-month and 1-year survival rates after CF-LVAD implantation for this group were 64.7% (11/17) and 58.8% (10/17), respectively (Fig. 1). The most common postoperative adverse events after CF-LVAD implantation were gastrointestinal bleeding (5/17, 29.4%), stroke (3/17, 17.6%), postoperative right heart failure requiring right ventricular assist device implantation (4/17, 23.5%) and sepsis (4/17, 23.5%).

**Figure 2:**
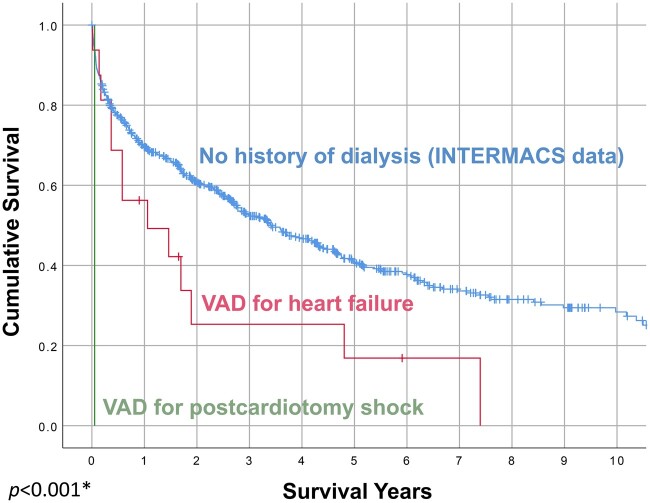
Survival after continuous-flow left ventricular assist device implantation, by ventricular assist device insertion indication. The survival curves are statistically different in log-rank analysis. The blue line represents survival in all patients from the Interagency Registry for Mechanically Assisted Circulatory Support registry implanted with a first continuous-flow left ventricular assist device during the same timeframe as our study; it is included for reference and was not compared statistically to the other survival curves. *P <* *0.001, log-rank test comparing ventricular assist device for heart failure versus ventricular assist device for postcardiotomy shock. INTERMACS: Interagency Registry for Mechanically Assisted Circulatory Support; VAD: ventricular assist device.

Two patients in the long-term dialysis group who died within the first 30 days had undergone emergency CF-LVAD implantation for postcardiotomy shock. These 2 patients were INTERMACS 1 preoperatively. If these emergency implant patients are excluded, the in-hospital mortality rate decreases to 20.0% (3/15), and the 6-month and 1-year survival rates are 73.3% (11/15) and 66.7% (10/15), respectively (Fig. 2).

#### Short-term dialysis group

In comparison with the long-term dialysis group, the short-term dialysis patients had poorer outcomes. All 14 short-term dialysis patients had the CF-LVAD implanted as a bridge to transplant. In-hospital mortality was much higher for this group than for the long-term dialysis group, at 85.7% (12/14; *P *=* *0.01). Only 2 patients survived to discharge after CF-LVAD implantation. Overall survival also was significantly worse, with 6-month and 1-year survival rates of 14.3% (2/14) and 7.1% (1/14), respectively (Fig. 1). None of these patients survived to heart transplant.

#### Group comparison

Patients stable on long-term dialysis had acceptable long-term survival and markedly better 6-month and 1-year survival by log-rank analysis than those who required short-term dialysis before implantation (64.7% vs 14.3% and 58.8% vs 7.1%, respectively; *P *<* *0.001; see Fig. 1).

## DISCUSSION

Increasingly, CF-LVADs are a common option for the expanding population of patients with end-stage heart failure. Although cardiac transplant, or even combined heart-kidney transplant, can be an ideal therapy for these patients, the complexity of combined heart-kidney transplant and the scarcity of donor hearts (exacerbated by the growing population of individuals diagnosed with heart failure) have resulted in an increasing reliance on mechanical circulatory support. An important goal of mechanical circulatory support is to bridge patients to cardiac transplant. However, multiorgan failure—including renal failure necessitating dialysis—develops in many candidates, limiting the possibility of a successful cardiac transplant.

Patients with renal failure who are being evaluated for CF-LVAD transplant present with 2 phenotypes. One group will have been stable on dialysis for months or years. The other group requires that dialysis be initiated in the days or weeks before CF-LVAD implantation. The challenges inherent to each group are different and unique. Fluid and electrolyte equilibrium is well established in patients on dialysis for months to years, whereas it may not yet have been achieved in patients who have only been on dialysis for days or weeks. Patients who have been on dialysis for months to years before undergoing CF-LVAD implantation primarily present with cardiac deterioration, whereas patients who have recently begun dialysis often have multiorgan problems. The differences in preoperative status between patients on dialysis for only days or weeks before CF-LVAD implantation versus those on dialysis for longer periods have important effects on surgical outcomes, perioperative management and patient survival, obligating a differentiation between these 2 patient groups. There are no clear definitions of ‘short-term’ and ‘long-term’ dialysis under standard classification schemes, such as the KDIGO (Kidney Disease Improving Global Outcomes), RIFLE (Risk, Injury, Failure, Loss of Kidney Function, and End-Stage Kidney Disease) or AKIN (Acute Kidney Injury Network) classifications [9–11]. Therefore, for the purposes of our analysis, we separated dialysis-dependent patients into those who began dialysis within 14 days of CF-LVAD implantation and those who had been on dialysis for >14 days before CF-LVAD implantation [12]. This separation into 2 distinct groups allows independent and clinically appropriate assessment of the 2 populations.

At our centre, the mortality rate among patients who are stable on maintenance dialysis for >14 days before CF-LVAD placement was markedly better than the rates reported by other authors [13]. It must be emphasized that our long-term dialysis patients were carefully selected and had few other comorbidities. Many patients on long-term dialysis are unsuitable for CF-LVAD implantation due to frailty, poor nutrition or other factors. The carefully selected patients in our study survived well beyond hospital discharge, with a median survival time of 1.3 years. At 1 year after CF-LVAD placement, 10/17 (58.8%) of the long-term dialysis group were living, including 1 patient who subsequently underwent a heart transplant. This 1-year survival rate is substantially lower than the 1-year survival rates of 81.1% and 82.1% reported in the INTERMACS registry for all patients who underwent placement of an axial-flow or centrifugal-flow CF-LVAD, respectively [4]. Nonetheless, 58.8% is a reasonable 1-year survival rate and is better than expected in this chronically ill patient cohort. In comparison, patients who required short-term dialysis before CF-LVAD placement have a distinctly worse prognosis: only 2 of our 14 patients (14.3%) survived to discharge.

Few reports have addressed CF-LVADs in patients with ESRD who require dialysis. Special challenges associated with these patients include strict attention to fluid management. Previous studies examining the relationship between preoperative kidney function and recovery after CF-LVAD implantation have focused on the general patient population or on patients with acute kidney injury [5–7, 14]. The few studies of patients with chronic renal failure requiring long-term dialysis have been limited to case reports [15, 16] and larger series based on data from administrative databases [13]. To our knowledge, ours is the largest observational study from a single institution.

In an analysis of the INTERMACS database, Kirklin *et al*. [13] found that the 85 patients who required preoperative dialysis before CF-LVAD implantation had a mortality rate of >30% in the first 3 months after implantation. The mortality rate approached 50% within the first 3 months for those patients who received preoperative dialysis and who were also INTERMACS class 1 before undergoing CF-LVAD implantation. However, because of the limitations of the INTERMACS database, the authors could not clarify whether the patients were receiving short-term or long-term dialysis.

In contrast, Bansal *et al*. [4] recently linked the United States Renal Data System database to Medicare claims to assess the outcomes of beneficiaries with ESRD who received their first CF-LVAD. A diagnosis of ESRD in the United States Renal Data System database depends on documentation of ESRD on the Center for Medicare and Medicaid Services Medical Evidence form and must be certified. Thus, the Bansal *et al.* sample is analogous to our long-term dialysis patients. In the Bansal *et al.* cohort, fewer than half of patients with ESRD at the time of CF-LVAD placement survived to hospital discharge, and the median survival time was only 16 days. By 1 year, 75.2% of patients with ESRD had died, and only 2.9% with a heart transplant were alive. The authors noted that they were the first to analyse the outcomes of patients on maintenance dialysis who underwent CF-LVAD implantation and that their results indicated a very poor prognosis for this patient population. Our observational data in patients with ESRD suggest a substantially better survival (as high as 66.7%) at 1 year when patients are carefully selected.

Our study and those by Kirklin *et al.* [13] and Bansal *et al.* [4] documented similarly poor survival rates with short-term dialysis. This is somewhat counterintuitive but probably reflects the fact that patients developing acute renal failure in the 14 days before CF-LVAD implantation are fairly unstable, compared with those patients who have been on dialysis for many months. It is also possible that some patients in the Bansal *et al.* [4] study were improperly coded as having ESRD for administrative purposes but actually had acute renal failure. If so, improper coding could account for the higher mortality rate seen in the Bansal *et al.* series.

Patients on dialysis for ≤14 days may represent the least haemodynamically optimized cohort, thus accounting for the high mortality observed in our short-term dialysis patients and in those in the Kirklin *et al.* [13] and Bansal *et al.* [4] studies. In our study, significantly more patients in the short-term dialysis group were INTERMACS 1, compared with the long-term dialysis group (64.3% vs 23.5%, respectively). Other demographic data did not correlate with outcomes in our study, and the incidence of ischaemic cardiomyopathy did not differ statistically between the short-term and long-term dialysis groups. These results indicate that haemodynamic stability plays an important role in determining which dialysis patients are appropriate candidates for CF-LVAD implantation.

The Medicare beneficiary database does not always allow for the analysis of indications for CF-LVAD implantation. Kirklin *et al*. [13] documented that INTERMACS class 1 patients have significantly worse survival. Our analysis of the long-term dialysis group also documented that those patients who underwent CF-LVAD implantation for postcardiotomy shock or as destination therapy had worse survival than those who received a CF-LVAD for progressive heart failure or as a bridge to transplant. In-hospital mortality was substantially lower at 29.4%, and 1-year survival was substantially higher at 58.8% in our series compared with results from the Kirklin *et al.* [13] and Bansal *et al.* [4] studies. When patients who underwent urgent CF-LVAD implantation for postcardiotomy shock (which carries a high mortality risk) were excluded, our in-hospital mortality rate decreased to 20.0% and the 1-year survival rate increased to 66.7%.

Bansal *et al*. [4] did not include details about reoperative CF-LVADs, and Kirklin *et al*. [13] did not specify whether patients were receiving a second CF-LVAD. We excluded patients with these conditions because the interplay between CF-LVAD physiology and renal function adds complicating factors to any analysis. Patients undergoing redo surgery for CF-LVAD exchange can have a higher mortality rate, particularly in the setting of renal failure. Including patients who have previously had an CF-LVAD may increase perioperative mortality.

Thus, our results are consistent with previous studies that may have included more diverse patient populations, including those at higher risk for mortality. Although our 1-year survival rate of 58.8% in patients on long-term dialysis (>14 days) was lower than the 1-year survival rate of 81.1% seen for axial-flow CF-LVADs in the INTERMACS registry [17], it is nonetheless an acceptable result. However, patients requiring short-term dialysis (≤14 days) before CF-LVAD placement had a survival rate of <15% and are not good candidates for CF-LVAD implantation. Haemodynamic instability as indicated by INTERMACS class was more common in the short-term dialysis group and is probably a marker of increased mortality. Because patients with haemodynamic instability have poor outcomes after CF-LVAD implantation, we are very hesitant to proceed with CF-LVAD implant in this population. Nevertheless, if careful attention is given to other clinical risk factors, CF-LVAD implantation can be done with acceptable morbidity and mortality outcomes in patients requiring long-term (>14 days) dialysis.

### Limitations

The limitations of this study include those inherent in any retrospective observational analysis. The power of the statistical analysis was limited, owing to the small number of patients with renal failure requiring dialysis before CF-LVAD implantation. Thus, the analysis cannot be adjusted for confounding factors. Given the small numbers of patients on dialysis undergoing CF-LVAD implantation worldwide, it is not possible to overcome this deficiency. Our limited, retrospective, single-centre study offers the best possibility for analysis.

HeartMate 3 CF-LVADs (Abbott, Abbott Park, IL, USA) were not included in our report. These devices were implanted under a clinical investigational protocol, MOMENTUM 3, at our institution after 2017. The protocol excluded patients who needed dialysis [18]. Therefore, no patients requiring dialysis underwent HeartMate 3 implantation.

As with the labels ‘short-term’ and ‘long-term’ renal failure, there are no clear definitions for ‘acute’ versus ‘chronic’ renal support. The need for renal replacement therapy for ≤14 days does not necessitate a diagnosis of ESRD. However, patients stable on dialysis for months or years present different surgical and clinical problems than patients whose dialysis was initiated days or weeks before CF-LVAD implantation. Although 14 days may seem an arbitrary cutoff for surgical decision-making, we found it to be clinically relevant.

Also of note, essentially all of the patients requiring short-term dialysis were INTERMACS 1 or 2, whereas only 70.6% of patients requiring long-term dialysis were INTERMACS 1 or 2. Finally, diagnostic measures such as renal histology or ultrasonography were not included in this study.

## CONCLUSION

Carefully selected patients who are stable on long-term dialysis can undergo CF-LVAD implantation for progressive heart failure, with acceptable morbidity and mortality. Patients on short-term dialysis before CF-LVAD implantation have a markedly worse prognosis and significantly poorer survival than do patients on maintenance dialysis for chronic ESRD. Patients with acute renal failure who have been on dialysis for ≤14 days and who have cardiogenic shock have a dismal prognosis and should not be considered for CF-LVAD placement.

Our results emphasize the importance of very careful patient selection and should be useful for counselling patients with ESRD and their families about outcomes after CF-LVAD implantation. CF-LVAD implantation is a reasonable option for select patients with chronic, stable ESRD and severe heart failure.

## ABBREVIATIONS

CF-LVAD: Continuous-flow left ventricular assist device
ESRD: End-stage renal disease
INTERMACS: Interagency Registry for Mechanically Assisted Circulatory Support
IQR: Interquartile range
